# CD69 enhances immunosuppressive function of regulatory T-cells and attenuates colitis by prompting IL-10 production

**DOI:** 10.1038/s41419-018-0927-9

**Published:** 2018-09-05

**Authors:** Lei Yu, Fei Yang, Fanghui Zhang, Danfeng Guo, Ling Li, Xian Wang, Tingbo Liang, Jianli Wang, Zhijian Cai, Hongchuan Jin

**Affiliations:** 1 Laboratory of Cancer Biology, The Key Lab of Biotherapy in Zhejiang Sir Run Run Shaw Hospital, Medical School of Zhejiang University, Hangzhou, China; 2 Institute of Immunology, and Bone Marrow Transplantation Center of the First Affiliated Hospital, Zhejiang University School of Medicine, Hangzhou, China; 3 Institute of Hematology, Zhejiang University & Zhejiang Engineering Laboratory for Stem Cell and Immunotherapy, Hangzhou, China; 4 Department of Nutrition and Food Hygiene, School of Public Health, School of Medicine, Zhejiang University, Hangzhou, China; 5 Chronic Disease Research Institute, School of Public Health, School of Medicine, Zhejiang University, Hangzhou, China; 6 Department of Medical Oncology, Sir Run Run Shaw Hospital, Medical School of Zhejiang University, Hangzhou, China; 7 Department of Hepatobiliary and Pancreatic Surgery, The Second Affiliated Hospital, Zhejiang University School of Medicine, Hangzhou, China

## Abstract

Foxp3^+^ regulatory T cells (Tregs) can inhibit immune responses and maintain immune tolerance by secreting immunosuppressive TGF-β1 and IL-10. However, the efficiency of Tregs become the major obstacle to their use for immunotherapy. In this study, we investigated the relevance of the C-type lectin receptor CD69 to the suppressive function. Compared to CD4^+^Foxp3^+^CD69^−^ Tregs (CD69^−^ Tregs), CD4^+^Foxp3^+^CD69^+^ Tregs (CD69^+^ Tregs) displayed stronger ability to maintain immune tolerance. CD69^+^ Tregs expressed higher levels of suppression-associated markers such as CTLA-4, ICOS, CD38 and GITR, and secreted higher levels of IL-10 but not TGF-β1. CD69^+^ Tregs from *Il10*^*+/+*^ rather than *Il10*^*−/−*^ mice significantly inhibit the proliferation of CD4^+^ T cells. CD69 over-expression stimulated higher levels of IL-10 and c-Maf expression, which was compromised by silencing of STAT3 or STAT5. In addition, the direct interaction of STAT3 with the c-Maf promoter was detected in cells with CD69 over-expression. Moreover, adoptive transfer of CD69^+^ Tregs but not CD69^−^Tregs or CD69^+^ Tregs deficient in IL-10 dramatically prevented the development of inflammatory bowel disease (IBD) in mice. Taken together, CD69 is important to the suppressive function of Tregs by promoting IL-10 production. CD69^+^ Tregs have the potential to develop new therapeutic approach for autoimmune diseases like IBD.

## Introduction

Tregs are very important in the maintenance of immune balance. During infection or inflammation, Treg cells can migrate from the blood to draining lymph nodes and inflamed tissues to inhibit the activation and proliferation of antigen-specific T-cells^1,2^. Tregs limit overwhelming immune response to pathogens via secretion of immunosuppressive cytokines such as IL-10 and TGF-β1. IL-10 inhibits both the proliferation and the cytokine synthesis of CD4^+^ T-cells^3,4^. IL-10 receptor-deficient Tregs failed to maintain Foxp3 expression and mice with deletion of IL-10 solely in Foxp3^+^ cells also develop inflammation in the intestine and elsewhere, demonstrating the relevance of IL-10 to immune tolerance^5,6^. TGF-β1 is known to promote Foxp3^+^ Treg cell generation. In both mice and humans, in vitro blockade of TGF-β1 through recombinant latency-associated peptide of TGF-β1 reverses the inhibitory effects of Tregs on CD4^+^ T-cell proliferation^7^. Moreover, a protective effect is achieved upon transferring wild-type CD4^+^CD25^+^ but not TGF-β1 deficient CD4^+^CD25^+^ T-cells in a severe combined immunodeficiency (SCID) model of colitis^7^. However, the real weight of TGF-β1 in controlling the magnitude of regulatory responses is still controversial, as recent works highlighted that deficiency of the TGF-β receptor on CD4^+^ T-cells induces a non-lethal form of colitis without leading to autoimmunity or multi-organ inflammation^8^.

Inflammatory bowel disease is thought to be caused by barrier disruption leading to the change in the intestinal flora and consequent activation of the mucosal immune system^9,10^. However, it is unknown whether the over-activated T-cells in IBD is the result of Treg function deficiency, resistance of T effector cells to suppression, or a combination of such two defects^11^. Adoptive transfer of Tregs can treat or prevent autoimmune diseases in animal models^12,13^. Unfortunately, Tregs purified from human blood do not consistently maintain Foxp3 expression and suppressive function^14^. In the presence of activated effector T-cells secreting inflammatory cytokines, mucosal tissues could preferentially shift Tregs towards Th17 cells to promote the pathogenesis of IBD^15,16^. Thus, it is very important to find suitable and effective Treg subsets in cellular therapeutics for autoimmune diseases.

Collective findings show that CD69 functions as a molecule involved in the regulation of immune response rather than a simple activation marker^17,18^. Naïve CD4 T-cells from CD69-deficient animals had a reduced ability to differentiate into Foxp3^+^ cells^19^. Moreover, CD69^+^CD4^+^ T-cells suppressed the production of proinflammatory cytokines by CD69^−^CD4^+^ T-cells in the murine model of spontaneous systemic lupus erythematosus^20^. Recent studies in CD69-deficient mice have revealed the role of CD69 in suppressing immune response through TGF-β^21,22^, CD69^+^CD4^+^CD25^−^ T-cells were confirmed to suppress T-cell proliferation through membrane-bound TGF-β1^23^. However, the function of IL-10 within the CD69^+^ Treg is largely unknown and still needs to be elucidated.

In this study, we investigated the relevance of CD69 to Tregs. There are two Treg subsets in mice, CD4^+^Foxp3^+^CD69^+^ and CD4^+^Foxp3^+^CD69^−^ Tregs. CD69^+^ Tregs were more potent to inactivate T cells. The differentiation of CD69^+^ Tregs to Th17 was also significantly reduced. In addition, CD69^+^ Tregs expressed higher levels of c-Maf to produce more immmuosuppressive IL-10. Interestingly, CD69^+^ Tregs but not CD69^−^ Tregs or *Il-10*-deficent CD69^+^ Tregs attenuated IBD in mice.

## Materials and methods

### Mice and cell lines

*Il10*^*−/*−^ C57BL/6 (H-2b) mice (stock number 004194) and *Rag1*^*−/−*^ (stock number 002096) mutant mice were purchased from the Jackson Laboratory. *Foxp3*^*GFP*^ knock-in C57BL/6 mice were generated by inserting the *GFP* gene into the endogenous *Foxp3* locus^24^ and were generously provided by Prof. Zhexiong Lian (University of Science and Technology of China). Female C57BL/6 at 6–16 weeks of age were from Joint Ventures Sipper BK Experimental Animal (Shanghai, China). All strains of mice were housed in a specific pathogen-free facility. The experimental protocols were approved by the Animal Care and Use Committee of Medical School of Zhejiang University (Hangzhou, China). Retroviral packaging cell line Platinum-E (Plat-E) and murine EL4 cell line were obtained from the American Type Culture Collection (ATCC, Rockville, USA).

### Isolation of CD69^+^ and CD69^−^ Tregs and CD4^+^CD62L^+^ naïve T-cells

Mononuclear cells suspensions were prepared from the spleen (Spl) and mesenteric lymph nodes (MLNs) of *Foxp3*^*GFP*^ knock-in mice, wild-type C57/BL6 mice, *Il10*^*+/+*^ or *Il10*^*−/−*^ mice. CD4^+^ T-cells were negatively selected using the CD4^+^ T-cell isolation Kit (STEMCELL) and a magnetic bead cell separator. Briefly, spleen and lymph node mononuclear cells were incubated with biotinylated antibodies against CD8, CD19, B220, MHC II, CD11c, IgM, DX5, and CD11b, and reacted with streptavidin microbeads, followed by separation with a magnetic cell separator. The flow-through CD4^+^ T-cells were collected and incubated with PE-anti-mouse CD69 (H1.2F3) or PE-Cy5- anti-CD69 (H1.2F3) and APC-anti-mouse CD4 antibody (GK1.5) or PE-Cy7-anti-mouse CD4 (RM4-5) for *Foxp3*^*GFP*^ knock-in mice, PE-anti-mouse CD69 (H1.2F3) or PE-Cy5- anti-mouse CD69(H1.2F3) and APC-anti-mouse CD4 (GK1.5) or PE-Cy7-anti-CD4 (RM4-5) as well as PE-anti-mouse CD25 antibody (CD25-4E3) for *Il10*^*−/−*^ mice and *Il10*^*+/+*^ mice, PE-cy7-anti-mouse CD4 (GK1.5) and eFluor-anti-mouse CD62L antibody (MEL-14) for wild-type mice. All of fluorescence antibodies were purchased from eBioscience. The FACSDiVa system (Becton Dickinson) was used to sort CD4^+^GFP^+^CD69^+^, CD4^+^GFP^+^CD69^−^, CD4^+^CD25^+^ CD69^+^, CD4^+^CD25^+^CD69^−^ Tregs and CD4^+^CD62L^+^ naïve T cells.

### RNA isolation, cDNA synthesis, and real-time PCR

Total RNA from the indicated types of cells was extracted with Trizol reagents (Invitrogen, Eugene, Oregon, USA) according to the manufacturers’ instruction. After being quantified by NanoDrop 2000 (Nanodrop, Wilmington, USA), the RNA samples were reversely transcribed into cDNA using the High Capacity cDNA Reverse Transcription Kit (Thermo fisher, Waltham, MA, USA). The relative levels of target gene mRNA transcripts were determined by real-time PCR using the SYBR Green Master Mix Kit (Thermo fisher). The sequences of primers are listed in Table 1. The PCR reactions were performed in duplicate at 94 °C for 30 s and subjected to 35 cycles of 94 °C for 15 s, and 60 °C for 20 s. Data analysis was done by using the comparative *C*_t_ method with β-actin as the normalization control.

**Table 1:** The sequences of primers

Gene	Forward primer	Reverse primer
*Real-time PCR primers*		
IL-10	5′-CCA AGC CTT GGA AA GA-3′	5′-TTT TCA CAG GGG AGA AAT CG-3′
c-Maf	5′-AGCAGTTGGTGACCATGTCG-3′	5′-TGGAGATCTCCTGCTTGAGG-3′
TGF-β1	5′-AACTGCACCCACTTCCCAGTC-3′	5′-CATTAAGGAGTCGGTTAGCAG-3′
T-bet	5′-CGTGGAGGTGAATGATGGA-3′	5′-TGG CAA AGG GGT TGT TGT CG-3′
GATA3	5′-GACTGAGAGAGCGAGACATAGA-3′	5′-AAGCAGACACGGAGGAATAAAG-3′
RORγt	5′-CCGCTGAGAGGGCTTCAC-3′	5′-TGCAGGAGTAGGCCACATTACA-3′
Foxp3	5′-CAGCTGCCTACAGTGCCCTAG-3′	5′-CATTTGCCAGCAGTGGGTAG-3′
Actin	5′-AACAGTCCGCCTAGAAGCAC-3′	5′-CGTTGACATCCGTAAAGACC-3′
CD69	5′-CCCTTGGGCTGTGTTAATAGTG-3′	5′- AACTTCTCGTACAAGCCTGGG-3′
*ChIP qPCR primers*		
STAT3	5′-AGGAGAAATACGAGAAGCTGGT-3′	5′-GGGAGAGGAAGGGTTGTCG-3′
SATA5b	5′-GGCCCAGGACTTGCAATTTT-3′	5′-CAAGACCGAGGTGCAGGC-3′
*Cloning PCR primes*		
CD69	5′-CCGGAATTCCAGGGACCTTGAGGGGAAAA-3′	5′-TTGCGGCCGCTCATCTGGAGGGCTTGCTGC-3′

### Western blotting analysis and ELISA

For western blot detection, crude proteins were extracted and the concentrations of proteins in individual cell lysate samples were determined using a Micro BCA protein assay kit (Thermo fisher). The cell lysates (30 µg/lane) were separated by sodium dodecyl sulfate polyacrylamide gel electrophoresis (SDS-PAGE) on 10–12% gels, and transferred to polyvinylidene difluoride (PVDF) membranes. After being blocked with 5% fat-free dry milk in TBST, the membranes were probed with primary antibodies, including anti-c-Maf (diluted 1:500), anti-p-STAT3 (diluted 1:500), anti-STAT3 (diluted 1:1000), anti-p-STAT5 (diluted 1:500), anti-STAT5 (diluted 1:500), and anti-β-actin (diluted 1:1000), all of these antibodies were from Cell Signaling Technology. The bound antibodies were detected with horseradish peroxidase (HRP)-conjugated secondary antibodies (Santa Cruz, CA, USA) and visualized by the enhanced chemiluminescent reagents (Thermo fisher). The levels of IL-17, IL-10, and TGF-β1 in the supernatants of cultured cells and IL-6, TNF-α, IFN-γ, and IL-17 in the serum of mice were measured by enzyme-linked immunosorbent assay (ELISA) using specific kits (eBioscience). For detection of TGF-β1, 100 μl of supernatants had been acidified with 20 μl 1N HCl for 10 min at room temperature and then neutralized with 20 μl 1N NaOH to activate latent TGFβ1 to the immunoreactive form.

### Tregs suppression assay

The suppression of two subsets of Tregs on CD4^+^ effector T-cell proliferation was determined by the CFSE incorporation assay. Murine splenic CD4^+^ cells isolated by a CD4^+^ T-cell isolation kit II (Miltenyi Biotec, Bergisch Gladbach, Germany) were labeled with CFSE (Invitrogen), according to the manufacturer’s instruction. Briefly, CFSE labeled CD4^+^ T-cells (1 × 10^6^/ml) were stimulated with 1 μl anti-CD3/CD28-coated beads (Invitrogen) with CD4^+^CD25^+^CD69^+^ Tregs, CD4^+^CD25^+^CD69^−^ Tregs in *Il10*^*+/+*^ mice or CD4^+^CD25^+^CD69^+^ and CD4^+^CD25^+^CD69^−^Tregs deficient in *Il10* at a ratio of 1: 1, 1: 2, or 1: 4. Three days later, the cells were harvested and the proliferation of effector CD4^+^ T-cells were analyzed using flow cytometry. The effector CD4^+^ T-cells alone served as the control.

### In vitro Th1 and Th17 cell differentiation

To induce Th1 cell differentiation in vitro, CD4^+^CD62L^+^ naïve T-cells, CD4^+^Foxp3^+^CD69^−^ or CD4^+^Foxp3^+^CD69^+^ Tregs were sorted from *Foxp3*^*GFP*^ knock-in mice with cocktails of IL-12 (10 ng/ml), anti-CD3/anti-CD28 (2 µg/ml of each), 10 µg/ml of anti-IL-4 (R&D systems). The CD4^+^CD62L^+^ naïve T-cells without IL-12 and IL-4 were regarded as negative control. Four days later, the cells were harvested and analyzed using flow cytometry.

To induce Th17 cell differentiation in vitro, CD4^+^CD62L^+^ naïve T-cells, CD69^−^ or CD69^+^ Tregs were mixed with murine bone marrow-derived dendritic cells (BMDCs) prepared as described previously^25^ at a ratio of 10:1, in the presence of IL-6 (6 ng/ml), TGF-β1 (3 ng/ml), anti-CD3/anti-CD28 (2 µg/ml of each), 10 µg/ml of anti-IFN-γ and anti-IL-4 (R&D systems). The CD4^+^CD62L^+^ naïve T-cells without IL-6 and TGF-β1 were regarded as negative control. Four days later, the cells were harvested and analyzed using flow cytometry.

### Cell staining and FACS analysis

Antibodies including APC-anti-CD4 (RM4-5) or PE-Cy7-anti-CD4 (RM4-5) and PE-anti-CD69 (H1.2F3) or PE-Cy5-anti-CD69 (H1.2F3), PE-anti-CTLA-4 (UC10-4B9), PE-anti-ICOS (C398.41), PE-anti-CD31 (390), PE-anti-CD38 (90), PE-anti-CD41 (MWReg30), PE-anti-CD44 (IM7), PE-anti-CD62L (MEL-14), PE-anti-CCR7 (4B12), PE-anti-CXCR4 (2B11), PE-anti-ICAM (HA58), PE-anti-GITR (DTA-1), PE-anti-GAPR (YGIC86), or isotype controls were used to analyze the phenotype of two subsets of Tregs from *Foxp3*^*GFP*^ knock-in C57BL/6 mice. All of these fluorescence antibodies except The PE-anti-Phospho-Stat3 (D3A7) and Phospho-Stat5 (D47E7) (Cell Signal Technology) were purchased from eBioscience. The expression levels of individual molecules in those Tregs were determined by flow cytometry.

To analyze the trans-differentiation of two subsets of Tregs to Th1 or Th17 cells, Tregs and control CD4^+^ T cells were stimulated with the cell stimulation cocktails (eBioscience) for 6 h and stained with PE-Cy7-anti-CD4 antibodies. The cells were fixed, permeabilized and intracellularly stained with PE-anti-IFN-γ (B27) APC-anti-IL-17 (eBio64DEC17) antibodies or isotype controls. The percentages of CD4^+^IFN-γ^+^ and CD4^+^IL-17^+^ cells were analyzed by flow cytometry.

Intraepithelial lymphocytes (IEL) and colonic lamina propria lymphocytes (LPL) were prepared as described previously[26]. The colonic LPL of each group of colitis were stimulated with the cell stimulation cocktails (eBioscience) for 6 h before detection of CD4^+^IFN-γ^+^ and CD4^+^IL-17^+^ T cells.

### Retroviral vector construction and transfection

The full open reading frame (ORF) of CD69 was amplified by PCR using specific primers as listed in Table 1 (Sangong Biotech, Shanghai, China). Subsequently, the CD69 ORF was cloned into the retroviral vector pMX containing internal ribosomal entry site-green fluorescent protein (IRES-GFP) to generate the plasmid of pMX-CD69. The pMX-CD69 and control pMX were transfected into Plat-E cells to generate retrovirus with pMX-CD69 or pMX, respectively. Retroviral transfection was performed as described previously^27,28^. Briefly, EL4 cells were transfected with retrovirus at MOI (multiplicity of infection) of 50 in the presence of 5 μg/ml polybrene (Millipore) for 48 h, then the cells were collected for further experiments.

### c-Maf, CD69, STAT3, and STAT5 knockdown in vitro

*c-Maf* siRNA, *CD69* siRNA, *STAT3* siRNA, *STAT5* siRNA, or scrambled siRNA (Ctrl Si) were synthesized by Genepharma (Shanghai, China). Target sequences for *c-Maf*: 5′-ACCCUUCCUCUCCCGAAUUTT-3′ (sense), 5′-GGCCAUGGAAUAUGUUAAUTT-3′ (antisense); *CD69*: 5′-CCAUGGACCAGUAUACAUTT-3′ (sense), 5′-AUGUAUACUGGUGCCAUGGTT-3′ (antisense); *STAT3*: 5′-CCCGCCAAAUUAAGAATT-3′ (sense), 5′-UUCUUAAUUUGUUGGCGGGTT-3′ (antisense); *STAT5*: 5′-GGAUGAGAGCAUGGAUGUUTT-3′ (sense), 5′-AACAUCCAUGCUCUCAUCCTT-3′ (antisense); Scrambled sequences for *c-Maf, CD69, STAT3*, and *STAT5*: 5′-UUCUCGAACGUGUCACGUTT-3′ (sense), 5′-ACGUGACACGUUCGGAGAATT-3′ (antisense). For siRNA transfection, EL4 cells (5 × 10^5^/well) were seeded in 24-wells plates overnight and transfected using Lipofectamine^TM^ RNAiMAX transfection reagent for 48 h (Invitrogen).

### Chromatin immunoprecipitation (ChIP)

The interaction of STAT3 and STAT5 with specific DNA sequences of c-Maf in pMX and pMX-CD69-infected EL4 cells was characterized by ChIP assays using the specific kit according to the manufacture’s protocol (Millipore). Briefly, pMX or pMX-CD69-infected EL4 cells were fixed with 1% formaldehyde for 10 min at 37 ℃ followed by sonication. The DNA fragments were incubated with antibodies at 4 °C overnight. Antibodies used were anti-STAT3, anti-STAT5 antibodies and rabbit IgG (Cell Signal Technology). Abs/chromatin complexes were precipitated using protein G agarose (Millipore) and then reverse the crosslinkling at 65 ℃ for 4 h. The presence of indicated DNA sequences was assessed by quantitative PCR using primers listed in Table 1.

### Induction and treatment of intestinal colitis

To induce colitis in mice, female C57BL/6 mice at 6 weeks of age were randomized and provided with 2.0% (w/v) dextran sulfate sodium (DSS, 40 kDs, MP Biomecicals, CA, USA) in drinking water for 10 days. The control mice received normal drinking water. Their body weights were monitored daily. For Tregs treatment, mice of two days post DSS-induction were intravenously injected with the purified CD4^+^CD25^+^CD69^+^ or CD4^+^CD25^+^CD69^*−*^ Tregs from *Il10*^*+/+*^ or *Il10*^*−/−*^ mice (1 × 10^6^ cells/mouse, purity of these cells >95%). Body weight changes and disease activity index (DAI) were recorded from the first day to the end of the study. The DAI is the sum of weight loss, rigidity of stool specimens and the extent of hematochezia^25^. At the end of the experiment, the large intestines of individual mice were dissected out and fixed in 10% phosphate-buffered formalin. The paraffin-embedded sections (5 µm) were stained with hematoxylin and eosin (H&E)^29^.

For T-cell transfer-induced colitis, naïve CD4^+^ CD45RB^hi^ T-cells from *Foxp3*^*GFP*^ knock-in mice were enriched (CD4^+^ T-Cell Isolation Kit; Miltenyi Biotec) and single-cell suspensions were stained with APC-anti-CD4 (GK1.5), and PE–anti-CD45RB (C363.16A), all from eBioscience, followed by cell sorting (FACSAriaII) (purification > 99%). Sex-matched *Rag-1*^*−/−*^ recipient mice received 5 × 10^5^ CD4^+^Foxp3^−^CD45RB^hi^ T-cells by intravenous (i.v.) injection, 1 × 10^6^ CD69^+^ Tregs or CD69^−^ Tregs were injected i.v. from the *Foxp3*^*GFP*^ knock-in mice 21 days later. At the end of the experiment, the large intestines of individual mice were dissected out and fixed in 10% phosphate-buffered formalin. The paraffin-embedded sections (5 µm) were stained with H&E.

### Confocal microscopy

Individual mice were randomized and injected intravenously with PBS or 1 × 10^6^ CD69^+^ Tregs from *Foxp3*^*GFP*^ knock-in mice in 200 µl PBS. Three or six hours later, the mice were killed and their large intestines were dissected out, washed, and embedded in OCT. The cryostat intestinal sections (8 μm) were stained with rabbit anti-CD69 and mouse anti-Foxp3 antibodies. After being washed, the sections were incubated with Alexa Fluor 568 and Alexa Fluor 488 labeled secondary antibodies (Invitrogen) and stained with 4,6-diamidino-2-phenylindole (DAPI). The fluorescent signals were examined under a confocal microscope (Olympus FluoView FV1000) and imaged using the Olympus FluoView version 1.4a viewer (Olympus).

### Statistical analysis

Data are expressed as the mean ± SD. The difference among the groups were analyzed by One-way ANOVA and Student’s *t*-test using GraphPad Prism 5. *P*-value of <0.05 was considered statistically significant.

## Results

### Characterization of CD69^+^ Tregs

**Fig. 1:**
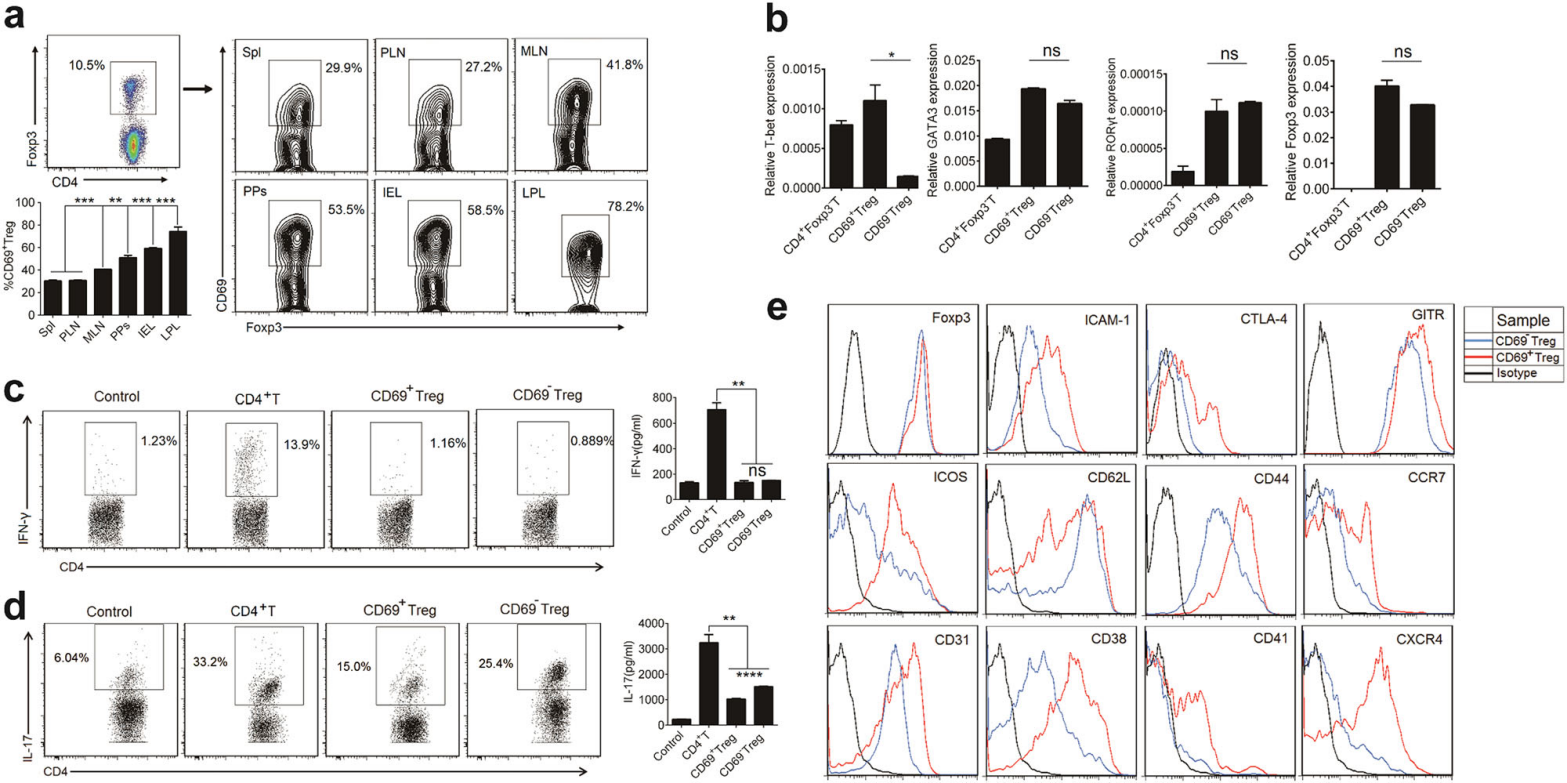
Characterization of CD4^+^Foxp3^+^CD69^+^ Tregs. **a** Density plots show CD69 expression in gated CD4^+^Foxp3^+^ cells from freshly isolated Spl, PLN and MLN, PPs, IEL, and colonic LPL in *Foxp3*^*GFP*^ knock-in mice. **b** Real-time PCR was performed to assess mRNA expression of *T-bet, GATA3, RORγt* and *Foxp3* genes in CD69^+^ Treg and CD69^−^Tregs. *β-actin* mRNA was used for the normalization. **c** 1 × 10^6^/ml naïve CD4^+^ T, CD69^+^ Treg or CD69^−^ Treg cells were cultured under Th1-cell-differentiation conditions for four days. The CD4^+^ naïve T-cells treatment without IL-12 were regarded as negative control. Each group of T-cells were stimulated with the cell stimulation cocktail for 6 h and then stained with anti-mouse CD4 and IFN-γ antibodies, followed by flow cytometry (left). The levels of IFN-γ in the supernatants of cultured T-cells were detected by ELISA (right). **d** BMDCs co-cultured respectively with 1 × 10^6^/ml naïve CD4^+^ T, CD69^+^ Treg, or CD69^−^Treg cells at a ratio of 1:5 under Th17-differentiation conditions for four days. The CD4+ naïve T-cells treatment without IL-6 and TGF-β1 were regarded as negative control. Each group of T-cells were stimulated with the cell stimulation cocktail for 6 h and then stained with anti-mouse CD4 and IL-17 antibodies, followed by flow cytometry (left). The levels of IL-17 in the supernatants of cultured T-cells were detected by ELISA (right). **e** The expression levels of immunosuppressive markers in CD69^+^ Treg and CD69^−^Tregs were analyzed using flow cytometry with indicated antibodies. Data are representative images or expressed as the mean ± SD of three independent experiments (*n* = 5). *P *<* 0.05, **P *<* 0.01, ****P *<* 0.0001, ns not significant, analyzed by ANOVA or Student’s *t*-test

To understand the distribution and possible biological relevance of CD69^+^ Tregs, we first analyzed CD4^+^Foxp3^+^CD69^+^ Tregs and CD4^+^Foxp3^+^CD69^−^Tregs from the spleen, MLN, peripheral lymph nodes (PLN, inguinal or axillary), Peyer’s patches (PPs), IEL and LPL in *Foxp3*^*GFP*^ knock-in C57BL/6 mice. Of total CD4^+^Foxp3^+^ Tregs, 25–30% in the spleen (Supplementary Fig. 1) or pLNs, 40–45% in the mLNs, 50–55% in PPs, about 60% in IEL and 75–80% in LPL were CD69^+^ Tregs (Fig. 1a). In addition, we found that expression level of transcription factor *T-bet* was higher in CD69^+^ Tregs, while no differential expression of *RORγt*, *GATA3* and *Foxp3* in two subsets were observed (Fig. 1b). Since T-bet contributes to maintain suppressive function of Tregs^30,31^, we then assessed functional difference between these two subsets of Tregs. First, we determined their potential to Th1 polarization. As shown in Fig. 1c, naïve CD4^+^ T cells were readily induced to produce IFN-γ under Th1-polarizing conditions. However, both the frequency of Th1 cells and IFN-γ production were similarly low in CD69^+^ Tregs and CD69^−^ Tregs under the same polarizing conditions, indicating that CD69^+^ Tregs and CD69^−^Tregs were resistant to acquire Th1 effector phenotype. Furthermore, both CD4^+^ T cells and CD69^−^ Tregs were readily induced to produce IL-17 under Th17-polarizing conditions. However, Th17 polarization was greatly compromised in CD69^+^ Tregs. Both the frequency of Th17 cells and IL-17 production were much less in CD69^+^ Tregs under the same polarizing conditions, indicating that CD69^+^ Tregs were resistant to acquire Th17 effector phenotype and might be more potent to inhibit immune response (Fig. 1d). Next, we compared the expression of immunosuppressive surface biomarkers^32–34^ in CD69^+^ Tregs and CD69^−^Tregs. Both subsets of Tregs expressed similar levels of Foxp3. However, CD44 and immunosuppression-associated markers such as CTLA-4, ICOS, CD38, and ICAM-1 were expressed at higher levels in CD69^+^ Tregs. In contrast, the expression of other markers including CD62L was lower in CD69^+^ Tregs (Fig. 1e). Hence, CD69^+^ Tregs had unique distribution in the mice and higher levels of immunosuppressive molecules expression, lower capacity to trans-differentiate into Th17 than CD69^−^Tregs.

### CD69^+^ Tregs produce more IL-10 to inhibit T cell proliferation

**Fig. 2:**
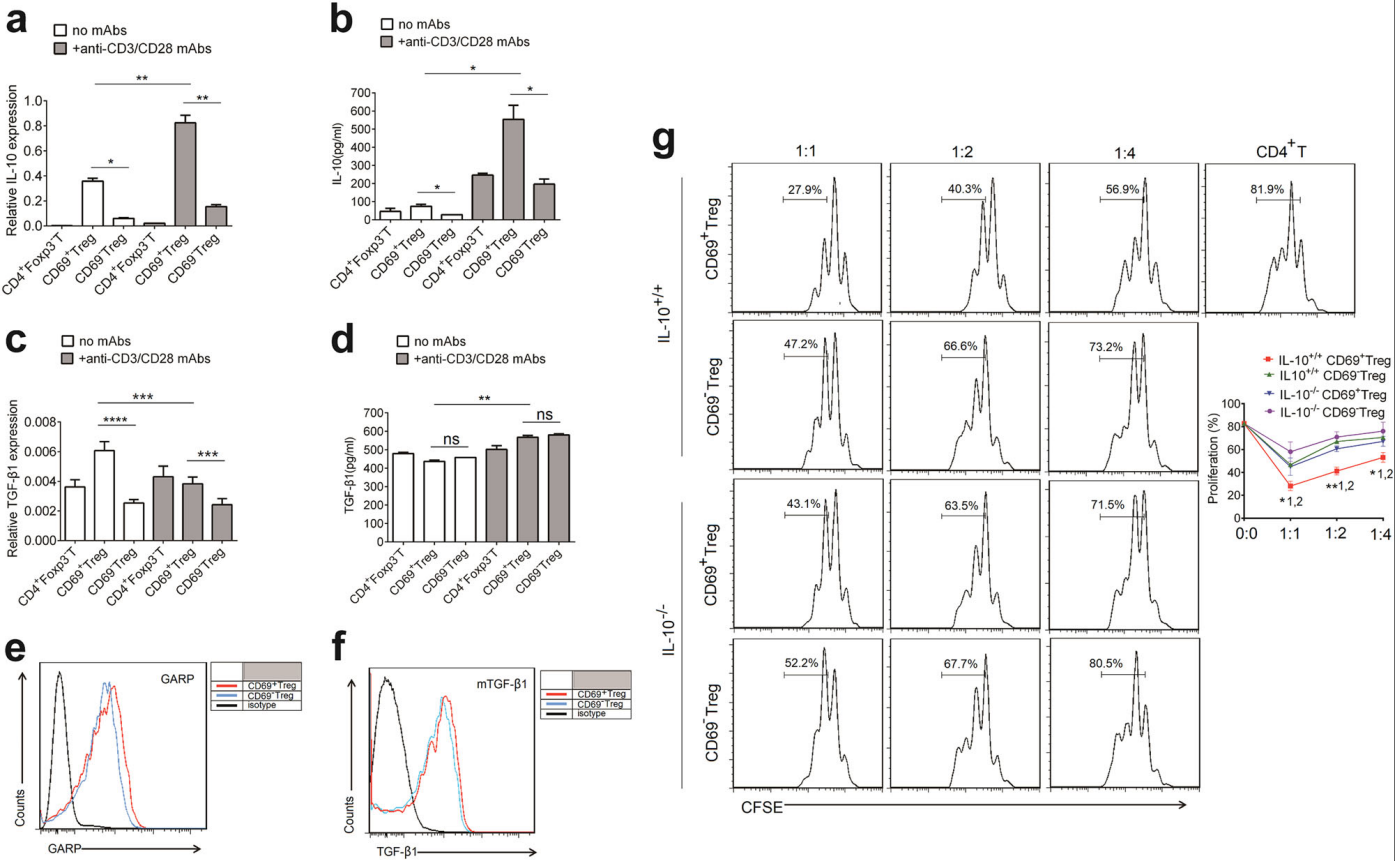
CD69^+^ Tregs inhibit effector T cell proliferation in an IL-10-dependent fashion. **a**, **c** Overall 1 × 10^6^/ml freshly isolated Treg cell subsets or CD4^+^Foxp3^−^T cells from *Foxp3*^*GFP*^ knock-in mice were stimulated with or without 2 μg/ml anti-CD3/CD28 antibody for 24 h and the relative levels of *Il-10* and *TGF-β1* to *β- actin* in the cells were analyzed by real-time PCR (*n* = 3). **b**, **d** The levels of IL-10 and TGF-β1 in the supernatants of cultured cells as described in **a** and **c** were measured by ELISA. **e**, **f** The expression levels of GARP and mTGF-β1 in CD69^+^ Treg and CD69^−^Tregs were analyzed using flow cytometry with indicated antibodies. **g** 1 × 10^6^ /ml CD4^+^CD25^−^T cells were labeled with CFSE and co-cultured with CD4^+^CD25^+^CD69^+^ Tregs or CD4^+^CD25^+^CD69^−^Tregs from *Il-10*^*+/+*^ or *Il-10*^*−/−*^ mice at a ratio of 1:1, 1:2, or 1:4 in the presence 1 μl anti-CD3/CD28 coated beads for three days. Then, the proliferation of CD4^+^ T cells was analyzed using flow cytometry. The cells were gated first on living lymphocytes and then CFSE^+^ T cells (*n* = 3). Date are representative images or expressed as the mean ± SD of three independent experiments, and Student’s *t*-test was used for statistical analysis. *P *<* 0.05, **P *<* 0.01, ***P *<* 0.001, ns, no significant. 1, versus CD69^−^ Tregs from *Il-10*^*+/+*^ mice; 2, versus CD69^+^ Tregs from *Il-10*^*−/−*^ mice

Tregs function to inhibit T-cell proliferation by mainly producing immunosuppressive cytokines, such as IL-10 and TGF-β1^35–37^. We therefore compared the capacity of two subsets of Tregs from *Foxp3*^*GFP*^ knock-in mice to produce such cytokines. We examined IL-10 and TGF-β1 production by freshly isolated resting T-cells including CD4^+^Foxp3^−^T cells, CD69^+^ Tregs and CD69^−^Tregs. As a result, both CD69^+^ and CD69^−^Tregs expressed higher level of *Il-10* mRNA than CD4^+^Foxp3^−^T cells. Interestingly, *Il-10* mRNA was six times more in CD69^+^ than in CD69^−^Tregs (Fig. 2a). Consistently, the secretion level of IL-10 protein were much higher from CD69^+^ Tregs (Fig. 2b). In contrast, although the level of *TGF-β1* mRNA were higher in CD69^+^ Treg than in CD69^−^Tregs, the relative protein levels of TGF-β1 in the supernatants were similar in all three types of T cells (Fig. 2c, d). We also compared the expression of both IL-10 and TGF-β1 in three subsets of activated T-cells. Activation of T-cells by anti-CD3/anti-CD28 antibodies effectively stimulated IL-10 expression in all three subsets (Fig. 2a–d). However, the levels of IL-10 but not TGF-β1 in the supernatants of CD69^+^ Tregs were much higher than these in the supernatants of other two types of T-cells. GARP (glycoprotein-A repetitions predominant, GARP) is a type I transmembrane cell surface docking receptor for latent TGF-β1 that is abundantly expressed on Tregs and platelets. GARP regulates the availability of membrane-bound latent TGF-β (mTGF-β1) and modulates its activation^38^. We found GARP was slightly higher in CD69^+^ Tregs than in CD69^−^Tregs, but mTGF-β1 had no difference between two subsets of Tregs (Fig. 2e, f).

Since CD69^+^ Tregs express the highest IL-10, we tested the role of IL-10 in suppressive function of CD69^+^Tregs. We isolated Tregs from either *Il-10*^*+/+*^ or *Il-10*^*−/−*^ mice and incubated them with CD4^+^ T cells from wild type mice. Interestingly, CD69^+^Tregs significantly inhibited CD4^+^ T cell proliferation in a dose-dependent manner, while CD69^−^Tregs had much weaker inhibitory effect (Fig. 2g). Moreover, the inhibitory effects of CD69^+^ Tregs were significantly compromised in the absence of IL-10. Together, these data suggested that CD69^+^ Tregs had a stronger ability to inhibit T-cell proliferation by producing higher level of IL-10.

### CD69 promotes IL-10 production

**Fig. 3:**
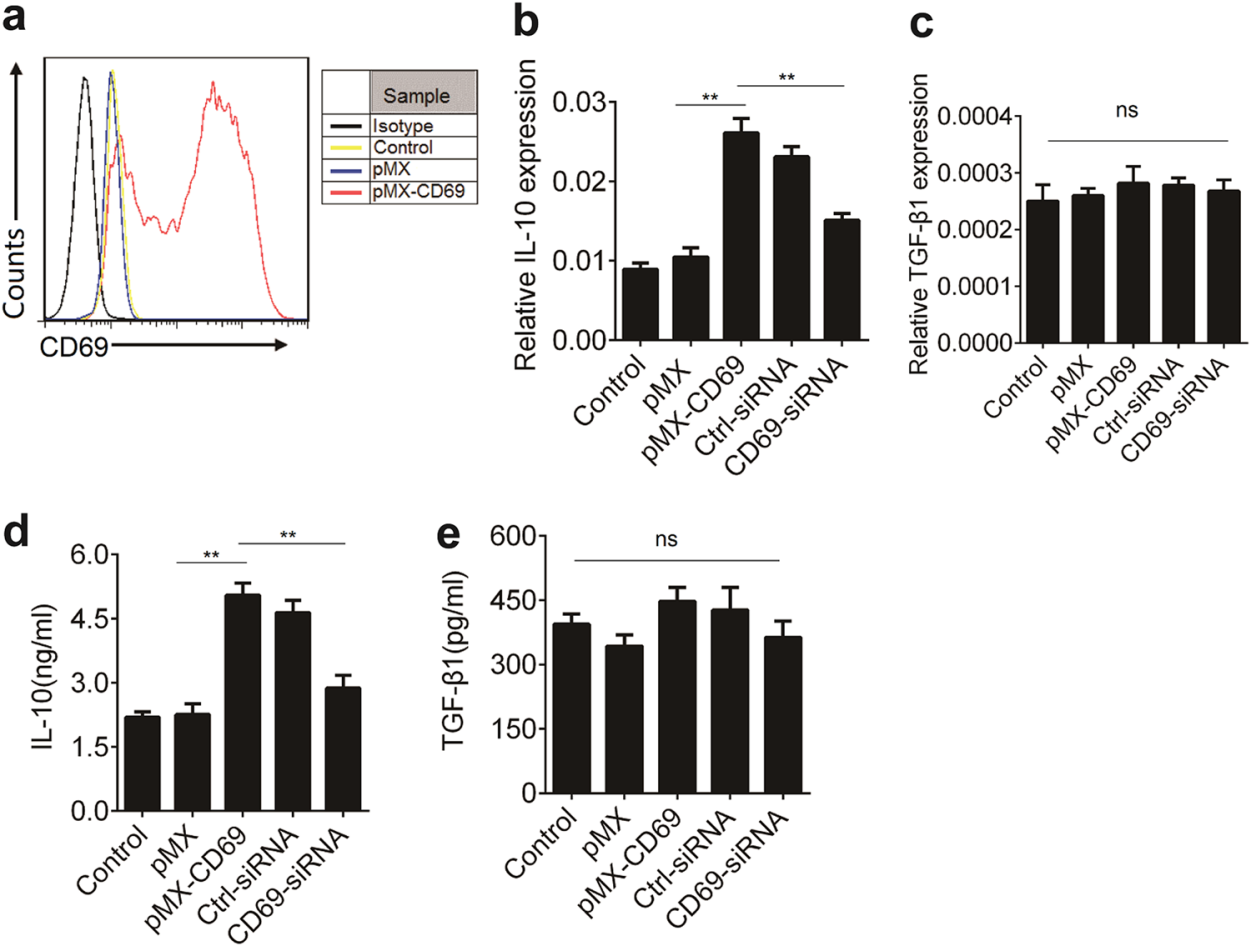
CD69 over-expression enhances IL-10 expression. 1 × 10^6^/ml EL4 cells were cultured in the supernatants from Plat-E cells that had transfected with control retrovirus pMX or pMX-CD69 at an MOI of 50 for 48 h. **a** The levels of CD69 were determined using flow cytometry. **b**, **c** *Il-10* and *TGF-β1* expression in EL4 cells treated as indicated were detected by real-time PCR (*n* = 3). **d**, **e** The levels of IL-10 and TGF-β1 in the supernatants of cultured cells as described in b and c were determined by ELISA (*n* = 3). Date are representative images or expressed as the mean ± SD of three independent experiments. Statistical significance is indicated by ANOVA or Student’s *t*-test. **P < 0.01, ns, no significant

To explore the potential mechanisms underlying the action of CD69 in promoting IL-10 production, we explored the effect of ectopic CD69 over-expression on IL-10 expression in EL4 cells. While EL4 cells expressed low amount of CD69 before retroviral infection, CD69 expression was remarkably increased after transfection (Fig. 3a). Next, we detected *Il10* and *TGF-β1* mRNA levels in EL4 cells before and after retroviral infection. We found that CD69 overexpression enhanced *Il-10* but not *TGF-β1* expression at transcript levels (Fig. 3b, c). Consistent with these results, CD69 overexpression increased the protein levels of IL-10 but not TGF-β1 (Fig. 3d, e). In contrast, knockdown of CD69 with *CD69* siRNA decreased IL-10 level but not TGF-β1 (Fig. 3b–e). Thus, CD69 was able to promote IL-10 transcription.

### CD69 promotes IL-10 production in a c-Maf-dependent manner

**Fig. 4:**
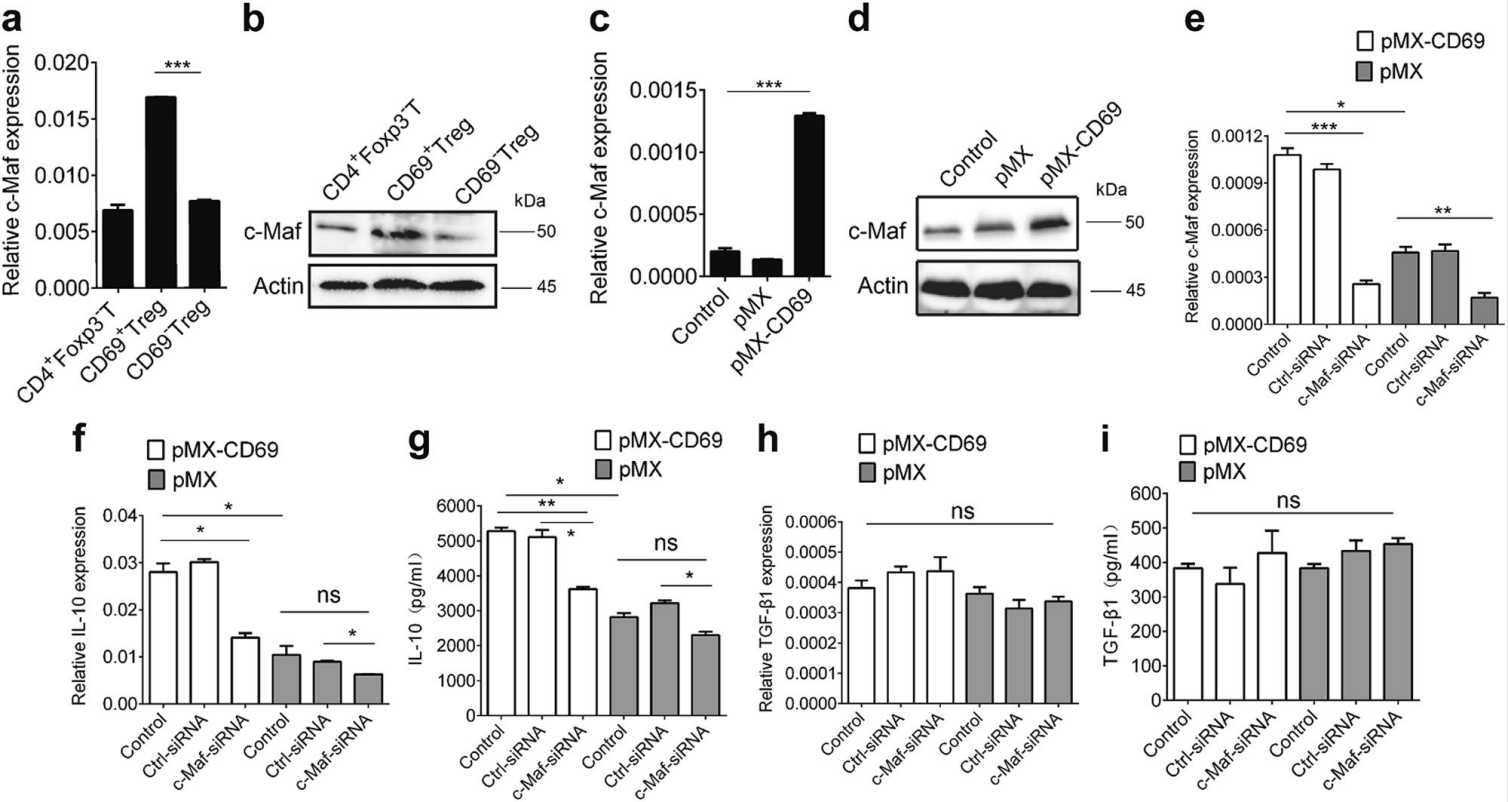
c-Maf is critical for CD69-induced IL-10 expression. **a**, **b** CD69^+^ Tregs, CD69^−^Tregs and CD4^+^Foxp3^−^T cells were isolated from *Foxp3*^*GFP*^ knock-in mice and the relative mRNA and protein levels of c-Maf in were analyzed by real-time RCR and western blot. **c**, **d** 1 × 10^6^/ml EL4 cells were infected with control retrovirus pMX or pMX-CD69 at MOI of 50 for 48 h to induce CD69 over-expression and the GFP positive cells were harvested. The relative mRNA and protein levels of c-Maf were analyzed by real time RCR and western blot. **e**–**i** The levels of c-Maf, IL-10, and TGF-β1 expression in CD69 overexpressing EL4 cells transfected with *c-Maf* siRNA or control siRNA were detected by real-time PCR and ELISA. Date are representative images or expressed as the mean ± SD of three independent experiments. *P < 0.05, **P < 0.01, ***P < 0.001, ns, no significant, ANOVA or Student’s *t*-test was used to determine significance

The transcription factor c-Maf is crucial for IL-10 expression^39,40^. Indeed, we found that the levels of *c-Maf* mRNA and protein in CD69^+^ Tregs were higher than in CD69^−^Tregs (Fig. 4a, b). Furthermore, CD69 over-expression in EL4 cells promoted *c-Maf* mRNA and protein expression (Fig. 4c, d). Moreover, both *Il10* mRNA and secreted protein levels were decreased once c-Maf expression was knocked down (Fig. 4e–g), while TGF-β1 levels were not altered (Fig. 4h, i). Therefore, CD69 promoted IL-10 expression in a c-Maf-dependent manner.

### STAT3 promotes IL-10 expression via c-Maf

**Fig. 5:**
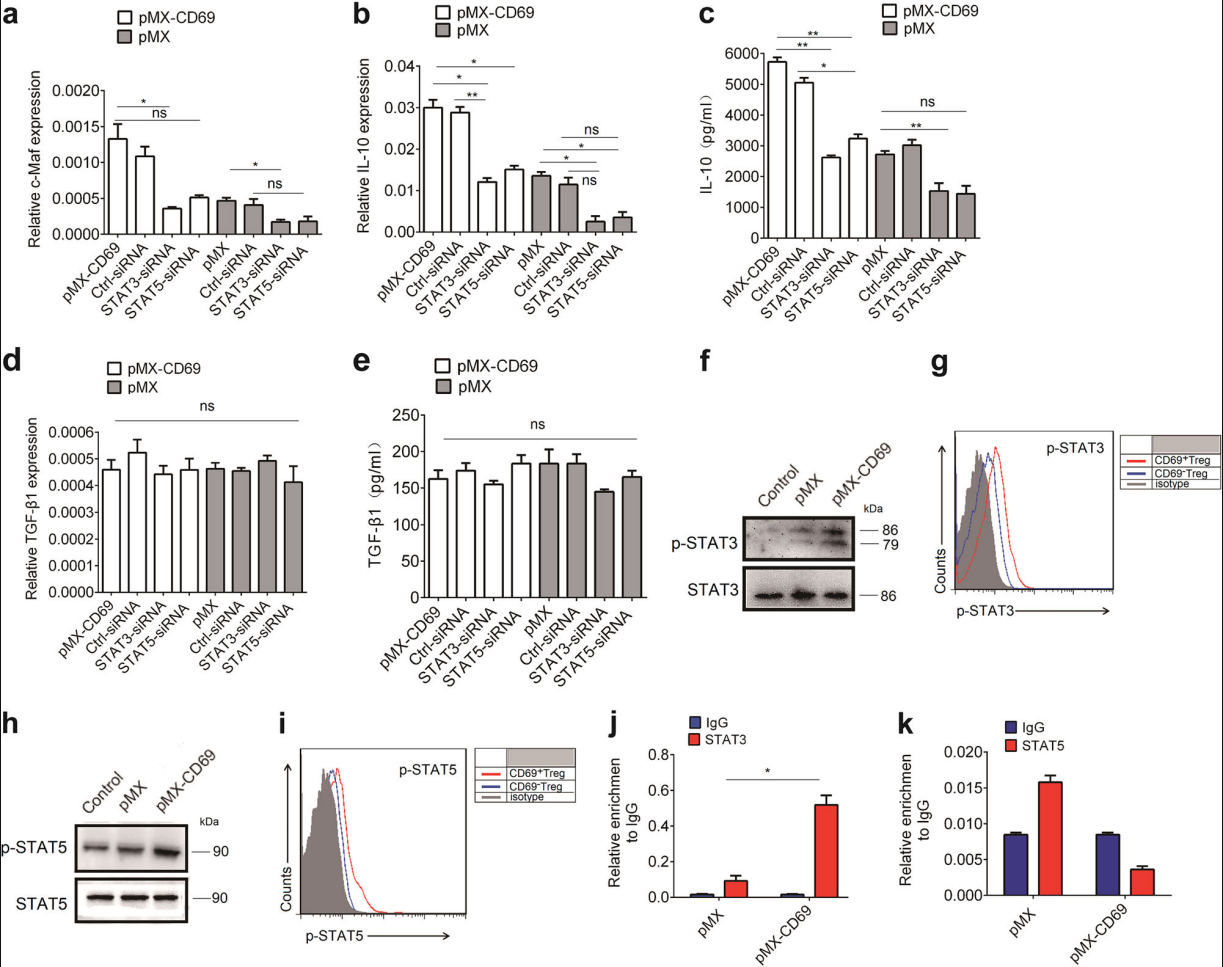
STAT3 is important to IL-10 expression induced by CD69. 1 × 10^6^/ml CD69^−^overexpressing EL4 cells were treated with *STAT3* siRNA or *STAT5* siRNA for the indicated time. The relative levels of c-Maf, IL-10, and TGF-β1 expression in the different groups of EL4 cells were determined by real time PCR and ELISA. **a** The mRNA level of *c-Maf* in pMX and pMX-CD69 transduced EL4 cells following treatment with *STAT3* siRNA or *STAT5* siRNA. **b**–**e** The mRNA and protein levels *IL-10* and *TGF-β1* expression in pMX and pMX-CD69 EL4 cells following treatment with *STAT3* siRNA or *STAT5* siRNA. **f**, **h** 1 × 10^6^/ml EL4 cells were transfected with control retrovirus pMX or pMX-CD69 to induce CD69 over-expression, GFP positive cells were harvested and the activation of STAT3 and STAT5 in CD69 over-expressed or pMX EL4 cells were measured by western blot. **g**, **i** CD4^+^Foxp3^+^CD69^+^ and CD4^+^Foxp3^+^CD69^−^ Tregs were fixed, permeabilized and intracellularly stained with anti-p-STAT3 or anti-p-STAT5 antibodies or isotype controls, then analyzed by flow cytometry. **j**, **k** ChIP analysis of STAT3 or STAT5 binding to the c-Maf promoter in CD69 over-expressing or control EL4 cells. Date are representative images or expressed as the mean ± SD of three independent experiments. *P < 0.05, **P < 0.01, ns, no significant, analyzed by ANOVA or Student’s *t*-test

Previous studies have shown that signal transducer and activator STAT3/STAT5 could enhance IL-10 transcription^41,42,43^. As c-Maf expression in CD69^+^ Tregs were higher than in CD69^−^Tregs (Fig. 4a, b), we investigated whether STAT3/STAT5 were involved in CD69-promoted c-Maf expression. As shown in Fig. 5a, treatment with either a *STAT3* or *STAT5* siRNA significantly reduced *c-Maf* mRNA levels in EL 4 cells with CD69 over-expression, accompanied by reduced IL-10 protein levels (Fig. 5b, c). Again, TGF-β1 expression was not altered (Fig. 5d, e). In addition, the phosphorylation of STAT3 and STAT5 was increased in CD69 over-expressed EL4 cells as well as CD69^+^Tregs (Fig. 5f–i). Furthermore, STAT3 was indeed able to interact with c-Maf promoter (Fig. 5j). However, to our surprise, we failed to detect the direct interaction of STAT5 with c-Maf promoter (Fig. 5k).

### Adoptive transfer of CD69^+^Tregs attenuated severity of chemical-induced IBD in mice

**Fig. 6:**
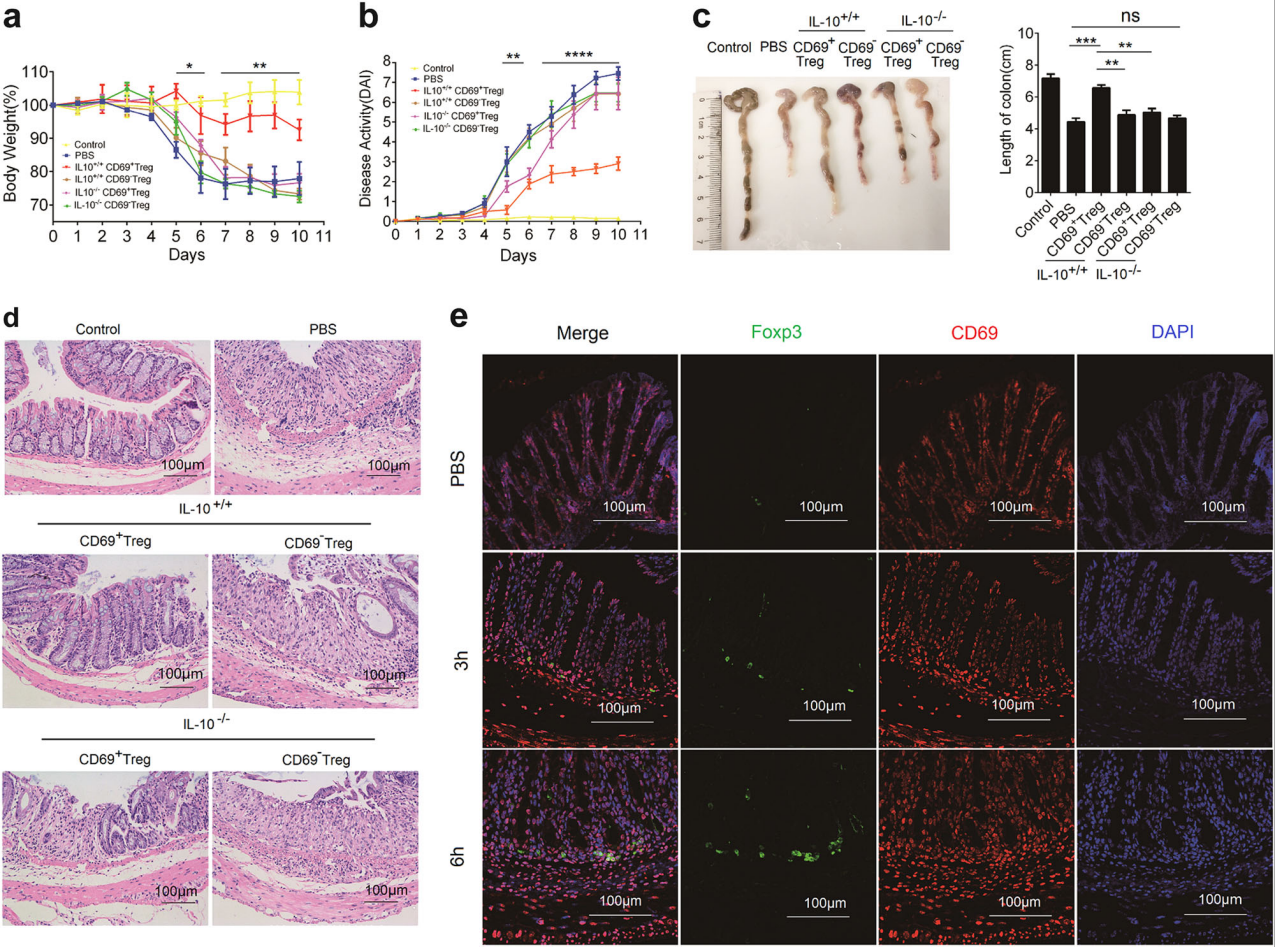
CD69^+^ Tregs attenuated DSS-induced IBD. IBD was induced by administering DSS in acidified drinking water, 2% (w/v) for 10 days. The day that mice started to drink DSS was regarded as day 0. For the treatment of IBD, 1 × 10^6^ CD4^+^CD25^+^CD69^+^ Tregs or CD4^+^CD25^+^CD69^-^ Tregs were sorted from the splenocytes of the *Il10*^*+/+*^ or *Il10*^*−/−*^mice and mice were injected i.v on day 2. **a** Body weight loss was recorded daily. Each point represents average weight data pooled from 8 mice ± SD. Control group: the mice fed with normal water; PBS group, mice with drinking water containing 2% DSS and intravenously treated with PBS on day 2. **b** Disease activity index (DAI, combined score of body weight, bleeding, and stool consistency) was evaluated daily. Each point represents average DAI data pooled from 8 mice. **c** Appearance and statistical analysis of colonic length on days 9. **d** Histological appearance of the colons from individual groups of mice 10 days after induction. The colonic sections were stained with H&E (magnification ×200). **e** Mouse received intravenous injection of 1 × 10^6^ /200 μl CD69^+^ Tregs or 200 μl PBS and then were killed 3 h or 6 h post injection. The colon tissues were dissected out and the cryostat colonic sections (8 μm) were stained with rabbit anti-CD69 and mouse anti-Foxp3 antibodies. The distribution of CD69^+^ Tregs in the colon tissues was detected with immunofluorescence assay. Date are representative images or expressed as the mean ± SD of three independent experiments (*n* = 7 per group). *P < 0.05, **P < 0.01, ***P < 0.001, ****P < 0.0001, analyzed by ANOVA or Student’s *t*-test

CD69^+^ Tregs were predominantly enriched in the MLN, PPs, IEL, and LPL (Fig. 1a), suggesting that CD69^+^ Tregs may play a role in intestinal immune balance. Therefore, we explored the relevance of CD69^+^ Tregs to intestinal inflammation in a mouse model of chemically induced colitis. Firstly, we detected the frequency of CD69^+^ Tregs in the thymus, spleen, MLN and PLN of mice at ages of 2, 4, 8, and 16 weeks, respectively. We found that CD69^+^ Tregs increased with age in the spleen and LNs, but not in the thymus (Supplementary Fig. 3a and b). Following induction of IBD, DSS induced loss of weight were also compromised with the increase of age (Supplementary Fig. 3c). In addition, the number and the ratio of CD69^+^ Tregs in the spleen and MLN were increased in a time-dependent manner (Supplementary Fig. 4). All of these results confirmed that CD69^+^ Tregs might play important roles in the maintenance of intestinal immune balance. Therefore, we examined the therapeutic efficiency of CD69^+^ Tregs in IBD mice. Two days after DSS induction, mice were randomized and intravenously injected with CD4^+^CD25^+^CD69^+^ Tregs or CD4^+^CD25^+^CD69^−^Tregs from *Il-10*^*+/+*^ mice or *Il-10*^*−/−*^ mice. Interestingly, treatment with CD69^+^ Tregs, but not CD69^−^Tregs or CD69^+^ Tregs deficient with IL-10 significantly attenuated DSS-induced loss of weight (Fig. 6a). Accordingly, DAI scores were also significantly reduced by the injection of CD69^+^ Tregs from WT mice but not *Il-10*^*−/−*^ mice (Fig. 6b). Similarly, injection with CD69^+^ Treg from WT mice but not from *Il-10*^*−/−*^ mice mitigated the DSS-induced shortening of colonic length in IBD mice (Fig. 6c). Histological examinations revealed dramatically decreased inflammatory infiltrates and less damage with greater conservation of the glandular structure in the colons of mice injected with CD69^+^ Tregs from *Il-10*^*+/+*^ mice (Fig. 6d). In addition, injected CD69^+^ Tregs tended to gradually traffic to the large intestine after 3 h i.v injection (Fig. 6e). Taken together, these data indicated that CD69^+^ Tregs suppressed chemical-induced colitis in a IL-10 dependent manner.

### CD69^+^ Tregs decreased the severity of murine T-cell transfer-induced colitis

**Fig. 7:**
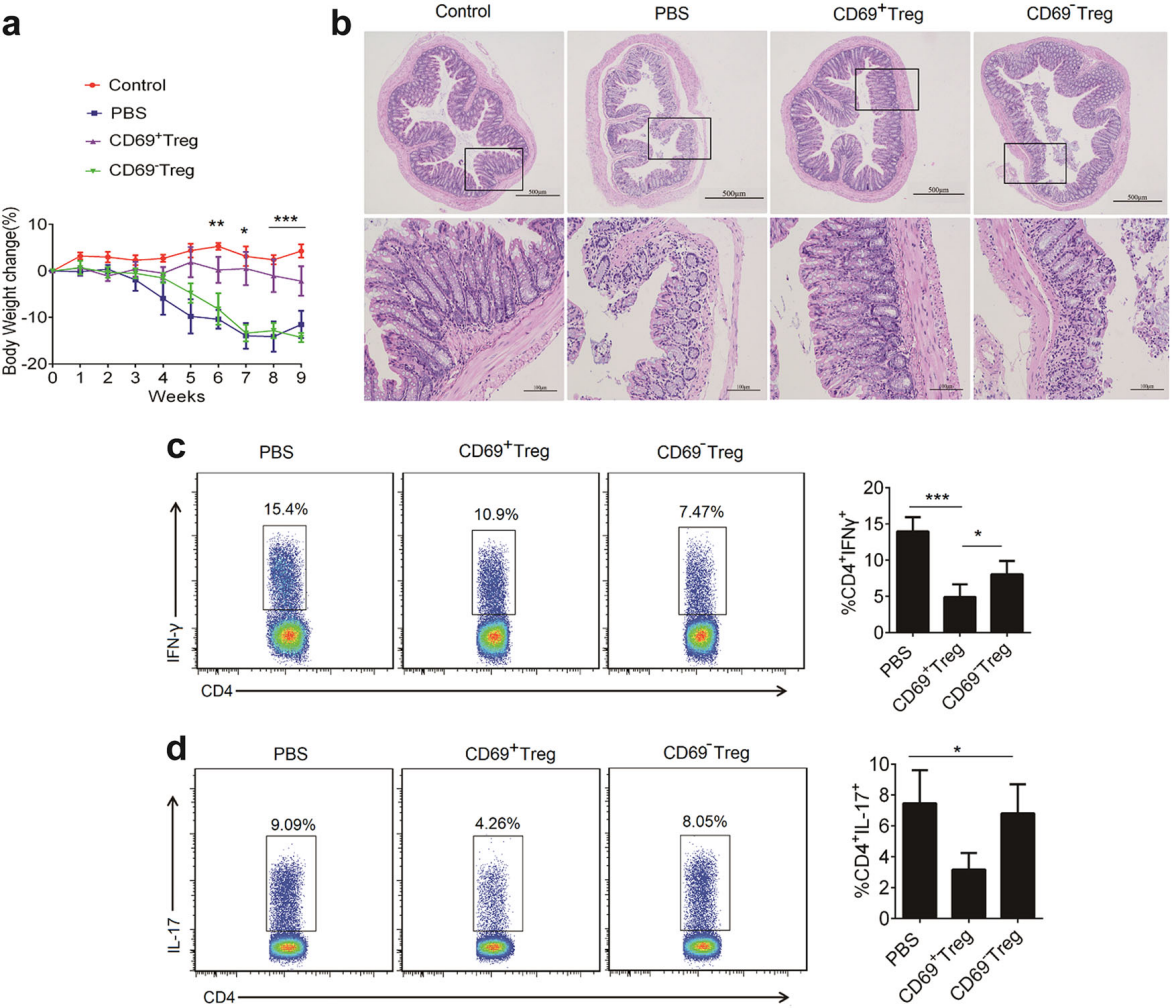
Adoptive transfer of CD69^+^ Tregs inhibits the disease progression in a mouse model of T-cell transfer-induced colitis. Spleen cells from *Foxp3*^*GFP*^ knock-in mice were enriched for CD4^+^ T-cells and then stained with anti-CD4 and anti-CD45RB monoclonal antibodies. Then CD4^+^Foxp3^−^CD45RB^hi^ T-cells were sorted and injected i.v into immunodeficient *Rag1*^*−/−*^ mice. Groups of mice were injected i.v with CD4^+^Foxp3^+^CD69^+^ Tregs and CD4^+^Foxp3^+^CD69^−^ Tregs from *Foxp3*^*GFP*^ knock-in mice (1 × 10^6^/mouse/injection) on days 21 (*n* = 7). **a** The body weights were measured for 9 weeks. Each point represents average weight data pooled from 7 mice ± SD. **b** Histological appearance 9 weeks after colitis induction. Representative colonic sections stained with H&E (Magnification: 40× and 200×). **c**, **d** Lamina propria mononuclear cells were isolated from colon of *Rag1*^*−/−*^ mice 8 weeks after the transfer of CD69^+^ Tregs and CD69^−^Tregs. The frequency Th1 and Th17 was detected by flow cytometry. Date are representative images or expressed as the mean ± SD of three independent experiments (*n* = 7 per group). *P < 0.05, **P < 0.01, analyzed by ANOVA and Student’s *t*-test

To further confirm the value of CD69^+^ Tregs in IBD treatment, we applied another model of colitis, T-cell transfer-induced colitis of *Rag1*^*−/−*^ mice. Twenty-one days after IBD induction, mice were intravenously injected with CD69^+^ Tregs or CD69^−^Tregs from *Foxp3*^*GFP*^ knock-in mice. Similar to chemical-induced IBD, T-cell transfer-induced colitis in *Rag1*^*-/-*^ mice were also attenuated by CD69^+^ Tregs but not CD69^−^Tregs (Fig. 7a). Histological assessment of colonic damages revealed that the mice untreated or received CD69^−^Treg suffered severe diffuse inflammation involving the mucosa, sub-mucosa, and in some cases extending through all intestinal layers. There was also pronounced disruption of the normal architecture and crypt loss. In contrast, mice treated with CD69^+^ Tregs had much less damage, showing more conserved glandular structure and limitation leukocyte infiltrations (Fig. 7b).

Th1 responses are crucial for the pathogenesis of IBD, while Th17 cells also contribute to the pathogenesis of IBD^9,10^. Therefore, Th1 and Th17 response were determined by flow cytometry. As shown in Fig. 7c, d, the frequency of Th1 and Th17 cells in CD69^+^ Tregs-treated mice were significantly less than that in PBS or CD69^−^Tregs treated mice. These results suggested that CD69^+^ Tregs possessed a potent immunoregulatory activity and were effective to inhibit the progression of T transfer colitis in *Rag1*^*−/−*^ mice.

## Discussion

Foxp3-expressing Tregs are crucial for peripheral immune tolerance by inhibiting harmful immune responses^44^. Despite a critical role for Treg cells in maintaining lympho-myeloid homeostasis, there is no reliable surface marker for isolation of living Tregs. In addition, it remains unclear how Treg cells exert their potent suppression functions. In previous studies, ICOS^+^Foxp3^+^ Tregs and ICOS^−^Foxp3^+^ Tregs were found in human thymus, peripheral blood and secondary lymphoid tissues^45^. In this study, we identified a unique subset of CD69^+^Tregs with potent inhibitory activity by producing high levels of IL-10. Compared with Foxp3^+^CD69^−^Tregs, Foxp3^+^CD69^+^ Tregs were more effective to suppress CD4^+^ T cell activity in an IL-10 dependent manner.

Studies in mice and human have shown that various activators such as type I IFN, phorbpl esters, anti-CD3 antibody can rapidly induce CD69 expression on T-cells in vitro or in vivo^17,19^. In human diseases, CD69 expression is increased on leukocytes at the site of inflammation^46^.CD69 is highly expressed by Tregs at mucosal sites indicating that CD69^+^Tregs may play essential role for the regulation of local inflammatory processes. Although CD69^−^Tregs also product IL-10 and TGF-β1 and express immunosuppressive molecule such as ICOS, GITR, and CTLA-4, they failed to attenuate DSS-induced colitis and T-cell transfer-induced colitis. It has been shown that Tregs can differentiate into effector cells because of “plasticity”. For example, in Th17-polarizing conditions, CD69^−^Tregs readily trans-differentiate into Th17 cells. As shown in Fig. 7, the frequency of CD4^+^IL-17^+^ T-cells were higher in CD69^−^Tregs treated mice, These findings suggest that CD69^−^Treg may be unstable under inflammatory conditions. Furthermore, studies showed that CD4^+^ T-cells accumulation in the murine colonic lamina propria during IBD is CD69 dependent, and the absence of CD69 deeply affected the pattern of chemokine stimulation by murine CD4^+^ T-cells^26^. Therefore, CD69 is most likely play an important role in Treg Function. However, how it regulates Tregs function remain largely unknown. Recently, CD69 was found to directly bind to S1P1 receptor on the lymphocyte surface, preventing the lymphocyte egress and prolonging T-cell retention^47^. Nevertheless, more studies are needed to clarify the function of CD69 in Tregs.

In addition, more questions remain open, such as the source of CD69^+^ Tregs. CD69 is expressed constitutively by thymus-derived regulatory T-cells and makes a substantial contribution to Treg cell development in the thymus^48^. However, we found that CD69^−^Tregs can be converted into the CD69^+^ Tregs upon stimulation with anti-CD3/CD28 antibodies (Supplementary Figure S2). In addition, the frequency of CD69^+^ Tregs in the spleen and LNs increased with age, but not in the thymus. Therefore, CD69^+^ Tregs may be also derived from the periphery organs and CD69^−^Tregs.

*CD69*^*−/−*^mice develop an exacerbated form of CIA (Collagen II-induced Arthritis) characterized by diminishing local synthesis of TGF-β1^21^. Another study suggests that CD69 is a negative regulator of the immune response, in part through modulation of local levels of TGF-β1^48^. However, our data indicated that the CD69^+^ Tregs were functionally dependent on IL-10. Studies showed that the cytoplasmatic domain of CD69 is associated with the Janus family kinase (Jak)3, which then activates the transcriptional factor STAT5^49^. However, the signaling cascade activated by CD69 is not defined in detail and the CD69 ligand is not be found yet. IL-10 is transregulated by a number of factors, including signal transducer and activator of transcription (STAT) molecules such as STAT3 and STAT5. We found that CD69 can increase the expression of both IL-10 and c-Maf. However, CD69-promoted IL-10 expression and secretion were compromised once c-Maf expression was knocked-down, supporting the notion that c-Maf is crucial for IL-10 expression. Therefore, STAT3/c-Maf signaling cascade could contribute to the increased IL-10 expression downstream of CD69. However, the relationship of STAT3 and STAT5 to CD69 still need be further explored.

In conclusion, our data indicated that CD69^+^Tregs had potent inhibitory activity against effector T-cell proliferation and inflammatory responses in an IL-10 dependent manner. CD69 may be critical for Tregs to maintain immune tolerance. The identification of CD69^+^Tregs may aid in the design of new therapies for the control of IBD and probably other autoimmune diseases.

## Electronic supplementary material

SUPPLEMENTAL FIGURES

## References

[1] Fontenot JD (2005). Regulatory T cell lineage specification by the forkhead transcription factor foxp3. Immunity.

[2] Hamano R (2014). Ag and IL-2 immune complexes efficiently expand Ag-specific Treg cells that migrate in response to chemokines and reduce localized immune responses. Eur. J. Immunol..

[3] Bai XF (1997). IL-10 suppresses experimental autoimmune neuritis and down-regulates TH1-type immune responses. Clin. Immunol. Immunopathol..

[4] Mosmann TR, Moore KW (1991). The role of IL-10 in crossregulation of TH1 and TH2 responses. Immunol. Today.

[5] Yang X, Gartner J, Zhu L, Wang S, Brunham RC (1999). IL-10 gene knockout mice show enhanced Th1-like protective immunity and absent granuloma formation following Chlamydia trachomatis lung infection. J. Immunol..

[6] Rubtsov YP (2008). Regulatory T cell-derived interleukin-10 limits inflammation at environmental interfaces. Immunity.

[7] Nakamura K (2004). TGF-beta 1 plays an important role in the mechanism of CD4+CD25+regulatory T cell activity in both humans and mice. J. Immunol..

[8] Sledzinska A (2013). TGF-beta signalling is required for CD4(+) T cell homeostasis but dispensable for regulatory T cell function. PLoS Biol..

[9] Danese S, Fiocchi C (2011). Ulcerative colitis. N. Engl. J. Med.

[10] Kaser A, Zeissig S, Blumberg RS (2010). Inflammatory bowel disease. Annu Rev. Immunol..

[11] Himmel ME, Yao Y, Orban PC, Steiner TS, Levings MK (2012). Regulatory T-cell therapy for inflammatory bowel disease: more questions than answers. Immunology.

[12] Hori S, Takahashi T, Sakaguchi S (2003). Control of autoimmunity by naturally arising regulatory CD4+T cells. Adv. Immunol..

[13] Adeegbe D, Bayer AL, Levy RB, Malek TR (2006). Cutting edge: allogeneic CD4+CD25+Foxp3+T regulatory cells suppress autoimmunity while establishing transplantation tolerance. J. Immunol..

[14] Murai M, Krause P, Cheroutre H, Kronenberg M (2010). Regulatory T-cell stability and plasticity in mucosal and systemic immune systems. Mucosal Immunol..

[15] Caprioli F, Pallone F, Monteleone G (2008). Th17 immune response in IBD: a new pathogenic mechanism. J. Crohns Colitis.

[16] Galvez J (2014). Role of Th17 cells in the pathogenesis of human IBD. ISRN Inflamm..

[17] De Maria R (1994). Triggering of human monocyte activation through CD69, a member of the natural killer cell gene complex family of signal transducing receptors. J. Exp. Med.

[18] Cibrian D, Sanchez-Madrid F (2017). CD69: from activation marker to metabolic gatekeeper. Eur. J. Immunol..

[19] Radulovic K (2012). CD69 regulates type I IFN-induced tolerogenic signals to mucosal CD4 T cells that attenuate their colitogenic potential. J. Immunol..

[20] Ishikawa S (1998). A subset of CD4+T cells expressing early activation antigen CD69 in murine lupus: possible abnormal regulatory role for cytokine imbalance. J. Immunol..

[21] Sancho D (2003). CD69 downregulates autoimmune reactivity through active transforming growth factor-beta production in collagen-induced arthritis. J. Clin. Invest..

[22] Radstake TR (2009). Increased frequency and compromised function of T regulatory cells in systemic sclerosis (SSc) is related to a diminished CD69 and TGFbeta expression. PLoS One.

[23] Han Y, Guo Q, Zhang M, Chen Z, Cao X (2009). CD69+CD4+CD25- T cells, a new subset of regulatory T cells, suppress T cell proliferation through membrane-bound TGF-beta 1. J. Immunol..

[24] Bettelli E (2006). Reciprocal developmental pathways for the generation of pathogenic effector TH17 and regulatory T cells. Nature.

[25] Cai Z (2010). TGF-beta1 gene-modified, immature dendritic cells delay the development of inflammatory bowel disease by inducing CD4(+)Foxp3(+) regulatory T cells. Cell Mol. Immunol..

[26] Radulovic K (2013). The early activation marker CD69 regulates the expression of chemokines and CD4 T cell accumulation in intestine. PLoS One.

[27] Logan GJ, Spinoulas A, Alexander SI, Smythe JA, Alexander IE (2004). CD4 expression on EL4 cells as an epiphenomenon of retroviral transduction and selection. Immunol. Cell Biol..

[28] Kitazawa M (2017). ASC induces apoptosis via activation of caspase-9 by enhancing gap junction-mediated intercellular communication. PLoS One.

[29] Aharoni R, Sonego H, Brenner O, Eilam R, Arnon R (2007). The therapeutic effect of glatiramer acetate in a murine model of inflammatory bowel disease is mediated by anti-inflammatory T-cells. Immunol. Lett..

[30] Xiong Y, Ahmad S, Iwami D, Brinkman CC, Bromberg JS (2016). T-bet regulates natural regulatory T cell afferent lymphatic migration and suppressive function. J. Immunol..

[31] Yu F, Sharma S, Edwards J, Feigenbaum L, Zhu J (2015). Dynamic expression of transcription factors T-bet and GATA-3 by regulatory T cells maintains immunotolerance. Nat. Immunol..

[32] Patton DT, Wilson MD, Rowan WC, Soond DR, Okkenhaug K (2011). The PI3K p110delta regulates expression of CD38 on regulatory T cells. PLoS One.

[33] Haas J (2007). Prevalence of newly generated naive regulatory T cells (Treg) is critical for Treg suppressive function and determines Treg dysfunction in multiple sclerosis. J. Immunol..

[34] Bollyky PL (2009). CD44 costimulation promotes FoxP3+regulatory T cell persistence and function via production of IL-2, IL-10, and TGF-beta. J. Immunol..

[35] Gordon KJ, Blobe GC (2008). Role of transforming growth factor-beta superfamily signaling pathways in human disease. Biochim Biophys. Acta.

[36] von Boehmer H (2005). Mechanisms of suppression by suppressor T cells. Nat. Immunol..

[37] Palomares O (2014). Regulatory T cells and immune regulation of allergic diseases: roles of IL-10 and TGF-beta. Genes Immun..

[38] Metelli A (2018). Immunoregulatory functions and the therapeutic implications of GARP-TGF-beta in inflammation and cancer. J. Hematol. Oncol..

[39] Xu J (2009). c-Maf regulates IL-10 expression during Th17 polarization. J. Immunol..

[40] Pot C (2009). Cutting edge: IL-27 induces the transcription factor c-Maf, cytokine IL-21, and the costimulatory receptor ICOS that coordinately act together to promote differentiation of IL-10-producing Tr1 cells. J. Immunol..

[41] Tsuji-Takayama K (2008). The production of IL-10 by human regulatory T cells is enhanced by IL-2 through a STAT5-responsive intronic enhancer in the IL-10 locus. J. Immunol..

[42] Tsuji-Takayama K (2008). IL-2 activation of STAT5 enhances production of IL-10 from human cytotoxic regulatory T cells, HOZOT. Exp. Hematol..

[43] Fontenot JD, Gavin MA, Rudensky AY (2003). Foxp3 programs the development and function of CD4+CD25+regulatory T cells. Nat. Immunol..

[44] Ito T (2008). Two functional subsets of FOXP3+regulatory T cells in human thymus and periphery. Immunity.

[45] Gonzalez-Amaro R, Cortes JR, Sanchez-Madrid F, Martin P (2013). Is CD69 an effective brake to control inflammatory diseases?. Trends Mol. Med.

[46] Shiow LR (2006). CD69 acts downstream of interferon-alpha/beta to inhibit S1P1 and lymphocyte egress from lymphoid organs. Nature.

[47] Cortes JR (2014). Maintenance of immune tolerance by Foxp3+regulatory T cells requires CD69 expression. J. Autoimmun..

[48] Martin P (2010). CD69 association with Jak3/Stat5 proteins regulates Th17 cell differentiation. Mol. Cell Biol..

[49] Hedrich, C. M. et al. Stat3 promotes IL-10 expression in lupus T cells through activation and chromatin remodeling . *Proc. Natl Acad. Sci. USA* **111**, 13457-13462 (2014) 10.1073/pnas.1408023111 PMC4169908 25187566

