# Supplementary material for: CD69 enhances immunosuppressive function of regulatory T-cells and attenuates colitis by prompting IL-10 production
Source: Cell Death Dis. 2018 Sep 5;9(9):905. doi: 10.1038/s41419-018-0927-9 (PMC6125584; doi:10.1038/s41419-018-0927-9)
Supplement: Supplementary file 1 — SUPPLEMENTAL FIGURES [file 41419_2018_927_MOESM1_ESM.pdf]

Fig.S1

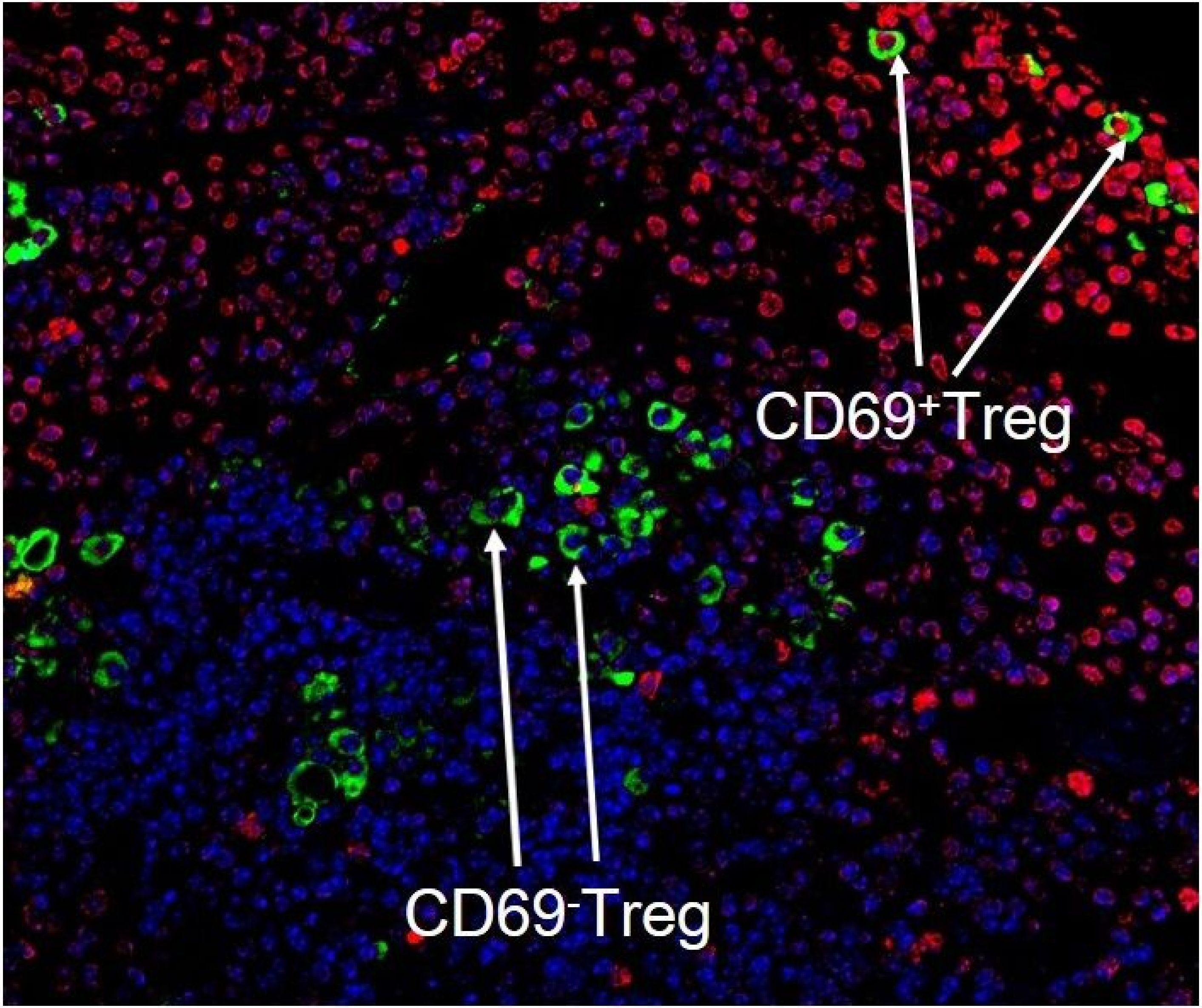

Fig.S2

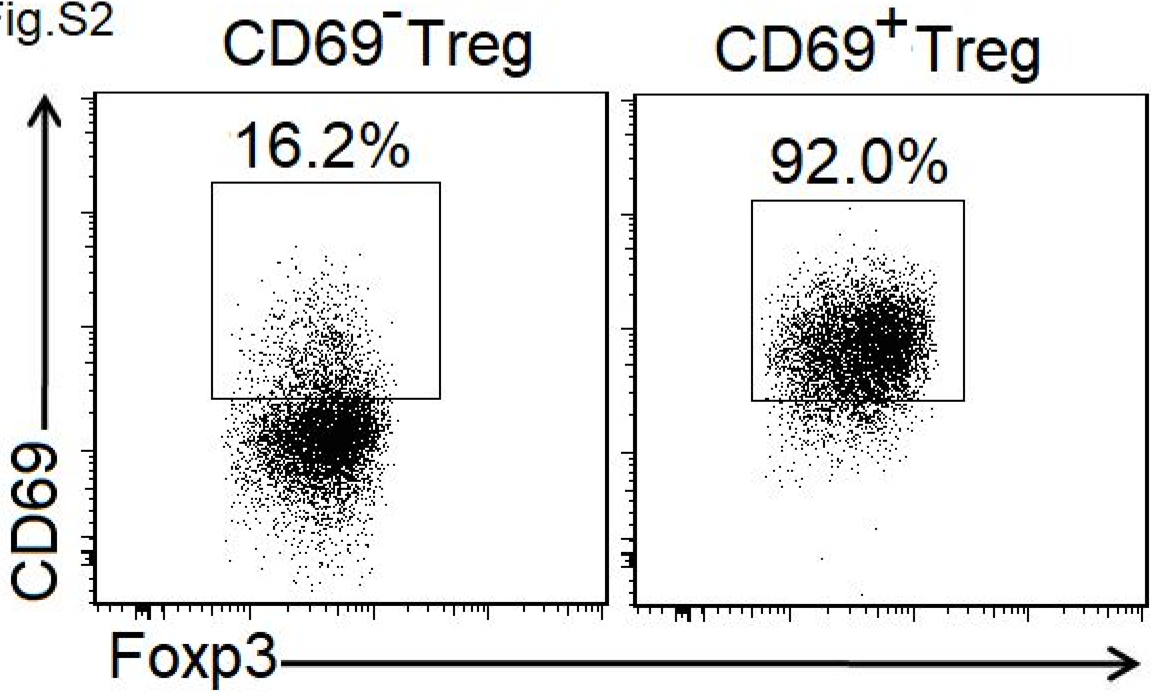

Fig.S3

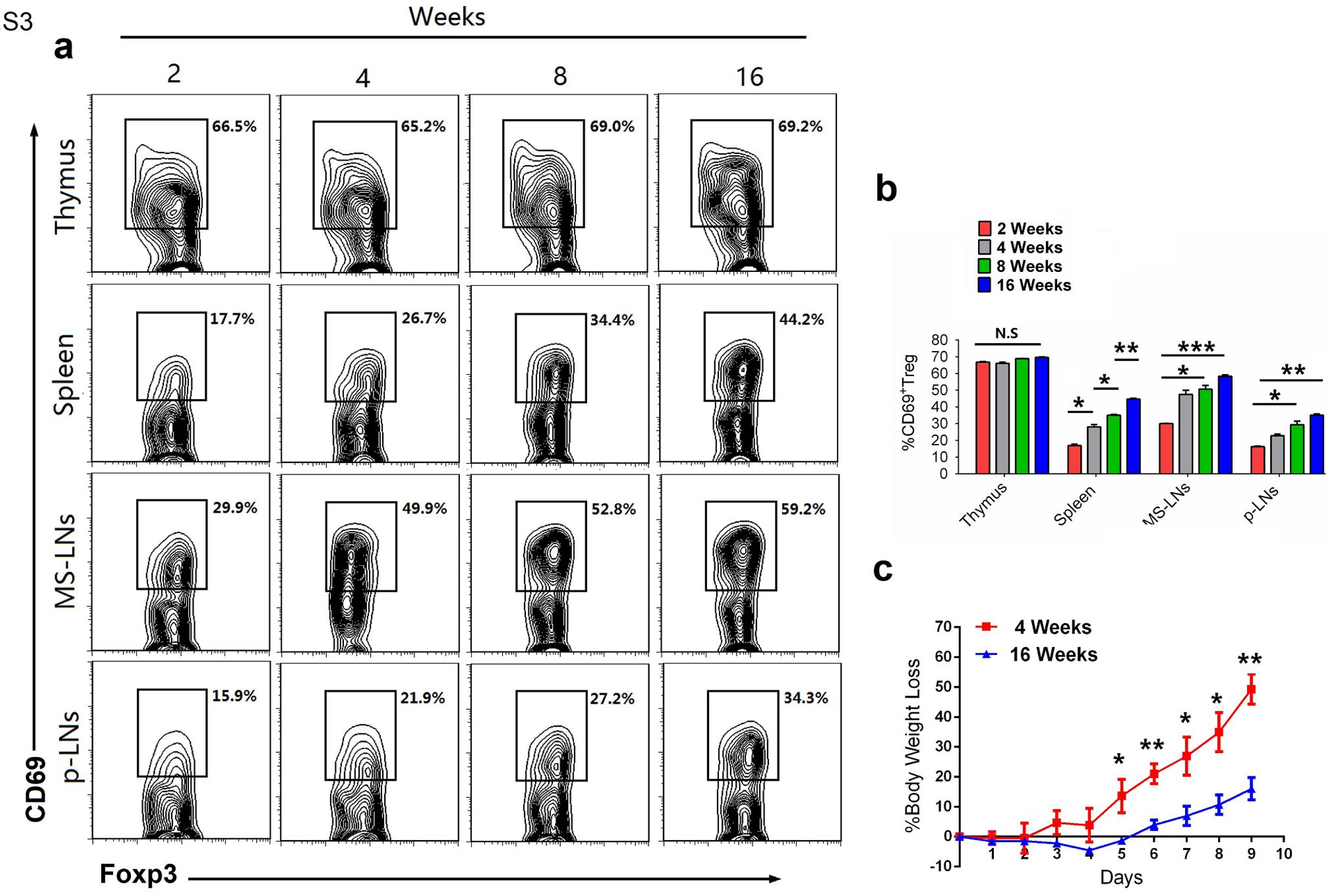
Weeks
a
2
4
8
16
Thymus
Spleen
MS-LNs
p-LNs
CD69
Foxp3
66.5%
65.2%
69.0%
69.2%
17.7%
26.7%
34.4%
44.2%
29.9%
49.9%
52.8%
59.2%
15.9%
21.9%
27.2%
34.3%
b
2 Weeks
4 Weeks
8 Weeks
16 Weeks
N.S
%CD69+Treg
Thymus
Spleen
MS-LNs
p-LNs
c
4 Weeks
16 Weeks
%Body Weight Loss
Days

Fig.S4

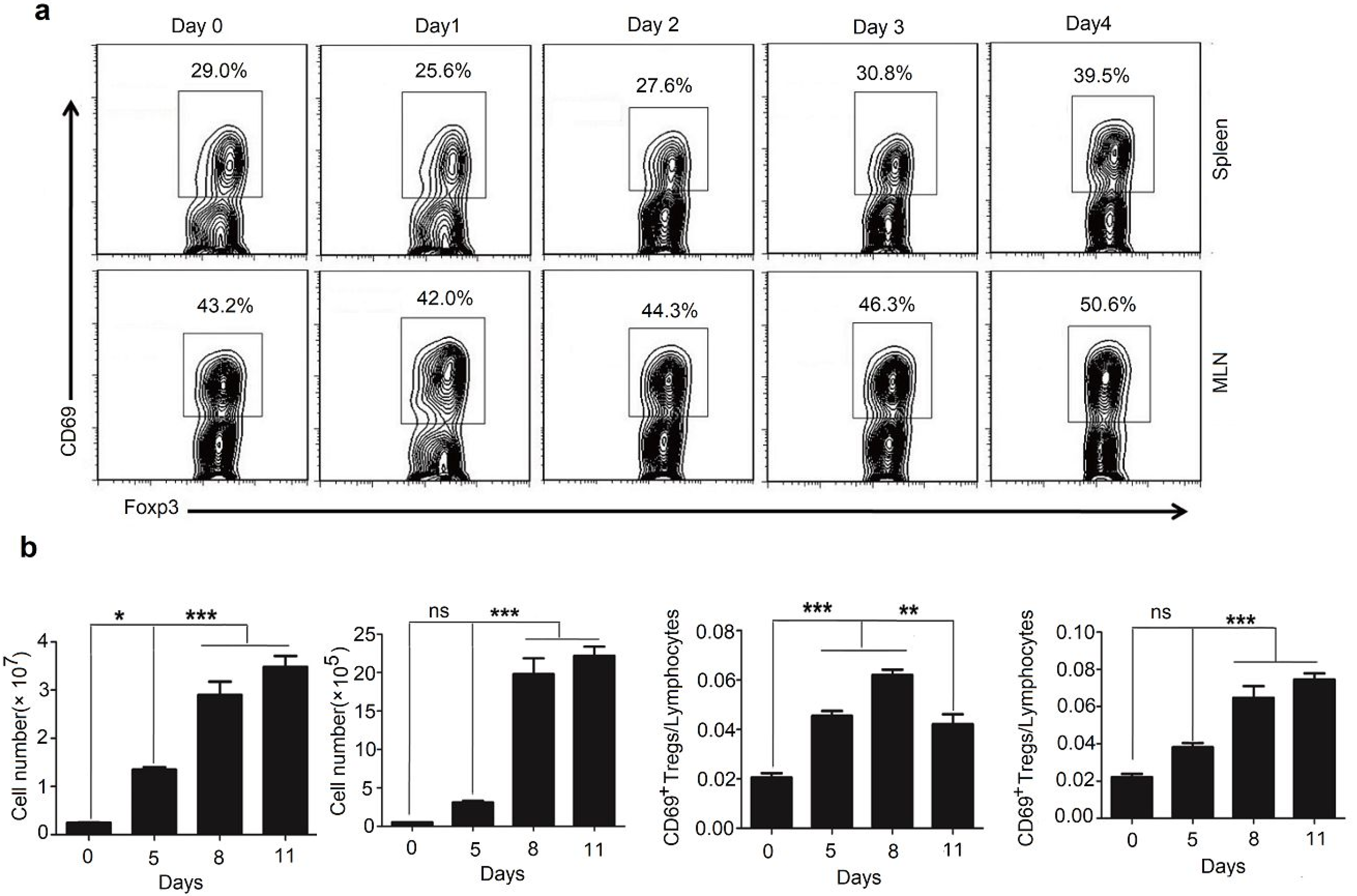

a
Day 0
Day1
Day 2
Day 3
Day4
29.0%
25.6%
27.6%
30.8%
39.5%
43.2%
42.0%
44.3%
46.3%
50.6%
Spleen
MLN
CD69
Foxp3
b
Cell number(× 10^7)
Cell number(×10^5)
CD69+ Tregs/Lymphocytes
CD69+ Tregs/Lymphocytes
Days
ns
*
**
***
0
5
8
11

Supplementary Figure Legends

Supplementary Figure. S1. $CD69^+$ Tregs and $CD69^-$ Tregs expression in spleen The spleens were dissected out and the cryostat sections (8 μm) were stained with rabbit anti-CD69 (red) and mouse anti-Foxp3 (green) antibodies. The distribution of $CD69^+$ Tregs in the spleen tissues was detection with immunofluorescence assay.

Supplementary Figure. S2 The CD69 is upregulated on $CD69^-$Treg upon activation .1×10^6/ml $CD69^+$ Tregs and $CD69^-$ Tregs were sorted from *Foxp3*^{GFP} knock-in mice and stimulated with 2μg/ml anti-CD3/CD28 antibodies for 48 h, the frequency of $CD4^+Foxp3^+CD69^+$ Tregs were analyzed by flow cytometry.

Supplementary Figure. S3. The relevance of $CD69^+$ Tregs to intestinal inflammation in different age of mice with chemically induced colitis (a-b)The percentages of $CD69^+$Tregs in the thymus, spleen and mLN and pLN of C57BL/6 mice at the indicated ages were characterized by flow cytometry.(c) Female C57BL/6 mice at 4 or 16 weeks of age were fed with 2% DSS solution (day 0) for 9 days to induced IBD. The body weights of individual mice were measured daily. Data are representative images or expressed as the mean ± SD of three independent experiments(n=10 per group).*$p<0.05$, **$p<0.01$, ***$p<0.001$, N.S, no significant, analyzed by ANOVA and Student's *t* test.

Supplementary Figure. S4. CD69$^+$ Tregs in progression of IBD

(a) After chemical induction, the frequency of CD69$^+$Tregc cells in spleen and mLN were detected by FACS. (b) The number and the ratio of CD69$^+$Treg in spleen and mLN increased after disease onset. Data are representative images or expressed as the mean ± SD of three independent experiments(n=10 per group).*p<0.05, **p<0.01， ***p<0.001, N.S， no significant, analyzed by ANOVA and Student's *t* test.
